# Understanding the relationship between age and information-seeking in the context of COVID-19

**DOI:** 10.1093/geroni/igaa057.3511

**Published:** 2020-12-16

**Authors:** Li Chu, Theresa Pauly, Elizabeth Zambrano Garza, Denis Gerstorf, Christiane Hoppmann

**Affiliations:** 1 The Chinese University of Hong Kong, Shatin, New Territories, China; 2 University of Zurich, Vancouver, British Columbia, Canada; 3 University of British Columbia, Vancouver, British Columbia, Canada; 4 Humboldt University Berlin, Berlin, Berlin, Germany

## Abstract

Socioemotional selectivity theory proposes that older adults engage in less information-seeking than younger adults as future time perspective becomes more limited and expansive goals are prioritized less. However, gathering information is crucial in emergencies like the COVID-19 pandemic, especially for older adults, who are particularly vulnerable to the virus. This study aims to better understand the association between age and information-seeking patterns during the current pandemic. Two hundred and sixty-six participants (age range = 18 – 84, Mage = 38.86, female = 77.06%, received postsecondary education = 83.08%, born in Canada = 73.68%) completed an online study between May and August 2020. We found that older age was associated with more information-seeking time (b = .45, SE = .16, p < .001). We then investigated whether perceived worries of getting COVID-19 might provide insights into this association. Findings point to a partial mediation with a significant direct effect (b = .37, SE = .16, p = .02, 95% bootstrap CI=[.07, .68]), a marginally significant indirect effect (b = .08, SE = .04, p = .06, 95% bootstrap CI=[-.003, .18]) and a significant total effect (b = .46, SE = .16, p < .001, 95% bootstrap CI=[.14, .77]). That is, older adults engaged in more information-seeking than younger adults in contexts in which information-seeking was personally relevant as indicated by perceived worries. These findings shed light on key correlates of information-seeking in older adulthood and highlight the importance for government and health organizations to make suitable information accessible for older adults.

